# Digital recording and documentation of endoscopic procedures: physicians’ practice and perspectives

**DOI:** 10.1186/s13584-019-0332-6

**Published:** 2019-07-02

**Authors:** Maya Peled-Raz, Nadav Willner, Dan Shteinberg, Keren Or-Chen, Tova Rainis

**Affiliations:** 1 The School of Public Health, The Center for Health, Law and Ethics, University of Haifa; Clinical Ethicist, Ethics Committee Chair, Bnai Zion Medical Center, Haifa, Israel; 2 Internal Ward B, Bnai Zion Medical Center, Faculty of Medicine, Technion-Israel Institute of Technology, 47 Golomb St, Haifa, Israel; 3 Bnai Zion Medical Center; Department of General Surgery, Bnai Zion Medical Center, Faculty of Medicine, Technion - Israel Institute of Technology, 47 Golomb St, Haifa, Israel; 4 School of Social Work, Faculty of Social Welfare & Health Science, University of Haifa, Mount Carmel, Haifa, Israel; 5 Head of Gastroenterology Unit Bnai Zion Medical Center, Faculty of Medicine, Technion-Israel Institute of Technology, 47 Golomb St, Haifa, Israel

**Keywords:** Digital Recording, Documentation, Physicians Survey, Endoscopic Procedures, Risk Management

## Abstract

**Background:**

In recent years, it has become increasingly prevalent internationally to record and archive digital recordings of endoscopic procedures. This emerging documentation tool raises weighty educational, ethical and legal issues – which are viewed as both deterrents and incentives to its adoption. We conducted a survey study aimed at evaluating the use of DRD in endoscopic procedures, to examine physicians’ support of this practice and to map the considerations weighed by physicians when deciding whether or not to support a more extensive use of DRD.

**Methods:**

Israeli physicians from specialties that employ endoscopic technics were surveyed anonymously for demographic background, existence and use of recording equipment, existence of institutional guidelines regarding DRD, and self-ranking (on a scale from 1 to 7) of personal attitudes regarding DRD.

**Results:**

322 physicians were surveyed. 84% reported performing routine endoscopic procedures, 78% had the required equipment for digital recording, and 64% of them stated that they never or only rarely actually recorded the procedure. General surgeons had the second highest rate of DRD equipment (96.5%) but the lowest rates of DRD practice (17.5%). The average ranking of support of DRD by all participants was 5.07 ± 1.9, indicating a moderately high level of support. Significant positive correlation exists between actual DRD rates and average support of DRD (*p* < 0.001). Based on mediation models, for all specialties and with no exceptions, having routine recording guidelines and positive support of DRD were found to increase the probability of actual recording. Being a surgeon or an urologist negatively correlated with support of DRD, and decreased actual recording rates. The argument “Recording might cause more lawsuits” was ranked significantly higher than all other arguments against DRD (*p* < 0.001), and “Recording could aid teaching of interns” was ranked higher than all other arguments in favor of DRD (*p* < 0.001).

**Conclusions:**

While DRD facilities and equipment are fairly widespread in Israel, the actual recording rate is generally low and varies among specialties. Having institutional guidelines requiring routine recording and a positive personal support of DRD correlated with actual DRD rates, with general surgeons being markedly less supportive of DRD and having the lowest actual recording rates. Physicians in all specialties were very much concerned about DRD’s potential to enhance lawsuits, and this greatly influenced their use of DRD.

These findings should be addressed by educational efforts, centering on professionals from reluctant specialties, as well as by the issuing of both professional and institutional guidelines endorsing DRD as well as requiring it where applicable.

**Electronic supplementary material:**

The online version of this article (10.1186/s13584-019-0332-6) contains supplementary material, which is available to authorized users.

## Introduction

The field of systematic digital recording and documentation (DRD) of endoscopic procedures is in its infancy. In Israel, many applicable activities and procedures are already video based. For example, procedures ranging from cardiac stent placement to arthroendoscopic surgery are performed using sophisticated video equipment; however, the record button is often turned off. The potential to harness the data in these videos and drive quality improvement may be substantial [1].

Advancement in technology and changes in societal perceptions have in recent years increasingly placed pressure to implement systematic video recording in the medical sphere. Most prominently, in the Netherlands, the Inspectorate of Health Care stated in a report on MIS that examination of the competency of laparoscopists based on national criteria is needed, as well as periodical assessment by colleagues via video recording of laparoscopic interventions [2].

The practice of DRD raises practical, ethical and legal issues that are frequently debated both in medical and legal writing [2–5]. Although video recording can be a powerful tool for various purposes – which will be reviewed in the next chapter, concerns have hampered implementation of systematic video recording. One concern is that videos invade the privacy of patients and professionals, by surveilling their activities and documenting their images [6]. In addition, professionals may fear that video data might be used for punitive or controlling purposes [2].

Our group has previously addressed the topic, exposing major differences between patients’ and physicians’ views of DRD. [7] DRD appears to be a double-edged sword: an exceptionally useful teaching and self-improving tool, but one that may prove harmful if used in the process of malpractice suit.

This survey study was aimed at evaluating the use of DRD in endoscopic procedures in Israel, examining physicians’ support of this practice and mapping the different considerations weighed when deciding whether or not to support a more extensive use of DRD.

## Recording of medical procedures in Israel – historical background

Recording of medical procedure was widely prevalent in medical institutions in Israel for many years, till it came to a screeching halt in 1995, in pursuance with the Israeli Supreme Court’s decision on the case of Hadassah Medical Center vs. Gilad [8]. In the Hadassah case the court was asked to grant a warrant, obligating the medical center to divulge the proceedings of an Internal Review Committee, assembled for the investigation of the successful suicide attempt of Mr. Gilad, on the hospital’s premises.

The Medical Center claimed that such proceeding should be categorized as confidential, so as to ensure the proper functioning of internal risk management and self-improvement operations, which in turn contributed profoundly to patients’ safety. This claim was rejected by the Supreme Court, which stated that the concern regarding the existence and functioning of teaching, self-improvement and risk management processes in hospitals, if confidentiality is not recognized, it is speculative, “…as these practices are in the essence of the medical profession, and of the ethical and legal commitments a person assumes when he undertakes to become a doctor.” “What is required from a doctor of medicine is not seclusion but transparency and disclosure of the truth,” so said the court.

Reality then proved the court wrong. The day after this ruling the Israeli Medical Association (IMA) issued a statement, warning physicians against the possible consequences of their participation in internal review committees. It also advised against the placing of cameras, meant to document medical care on trauma wards in order to draw conclusions from treatment mistakes and improve the teaching of interns – stating their fear that the tapes would be used as evidence in negligence lawsuits. This marked the effective elimination –not limited to trauma wards –of both those practices from most hospitals in Israel for years to come.

It has taken nearly two decades for the pendulum to start swinging back and for the use of digital recording for teaching and risk-management purposes to reappear in Israeli hospitals. Yet, concerns about the consequences of DRD are prevalent.

## The potential benefits of DRD

The current world wide literature points to three central potential benefits of systematic video recording of medical procedures:

### Monitoring processes for the purpose of improving quality, efficiency and safety of care

Receiving real-time feedback from video recordings has been shown to bear the long-term benefit of improving medical performance and thus patient safety and outcomes. Review of video-recordings has been found useful in improving performance of clinical skills, [9] cardiopulmonary resuscitation, [10] trauma resuscitation, [11] surgical procedures, [12] angiographic procedures, [13] and management of anesthetic crises [14].

In the event of complications and in the high-pressure operating room (OR) environment, it may be difficult for junior surgeons to appreciate what went wrong and how that could be avoided in the future. Having a video recording that can be reviewed at a later stage, with input from the supervisor in a safe environment, is a useful method for making the most of these situations.

Skills and team performance can be optimized by giving feedback through video images of intraoperative care. Just as football teams watch game tapes on Monday, an OR team could see how they functioned as a team, and this can lead to an appreciation of how seemingly inconsequential events and activities in the OR impact performance and influence outcomes [15].

Peer review of videos can also enhance existing quality improvement efforts [16]. For example, procedure videos can better inform morbidity and mortality conferences and sentinel event root-cause analyses that have traditionally relied on the notes of clinicians, which can be limited and even biased. Moreover, the exportability of video files can facilitate external review, allowing a peer reviewer removed from a local department’s politics to advise on what could have improved [1, 17].

Video recording has also been shown to impact the quality of care merely due the care-takers awareness of its existence [18, 19].

### Education of students and young professionals

There is an increased interest in real time digital recording as part of the teaching of surgical trainees and there is evidence that watching other surgeon operate can lead to an improvement in surgical outcomes [20].

Real time digital recording of procedures for teaching purposes is believed to ultimately lead to a better advanced surgical training experience [21], greater skill acquisition [22] and a reduction in the potential for surgical errors [23].

In the case of endoscopic procedures, the importance of systematic video recording of medical procedures is particularly great, as the nature of the procedure makes it less possible for trainees to learn by assisting, as they might in other branches of surgery and care [24].

Moreover, unusual or ground-breaking procedures may be recorded and disseminated so that other clinicians may view them and increase their knowledge base. Intraoperative complications can also be brought to a wider audience, so that lessons can be learned and safety improved for future patients [25].

### Better follow-ups

Saving a video of the procedure could be valuable for future physicians when treating a patient. Surgeons may benefit by watching a patient’s archived video of their last procedure. A physician’s operative note in the patient’s chart is often brief and does not capture the details of a video [1].

## Research methods

A total of 322 Israeli physicians were anonymously surveyed through their respective professional association using an online link, via the Google Docs platform. The survey included questions regarding their specialty, primary employment venue (hospital vs. community), existence of recording equipment, existence of institutional guidelines regarding DRD, and finally, their personal attitudes and common practice regarding DRD.

Physicians’ reasoning for and against DRD were evaluated based on their agreement with relevant statements, chosen based on a preliminary round-table expert discussion. For most questions, a scale from one to seven was used, with one being “absolutely disagree” and seven being “absolutely agree.” Differences between rankings in two level variables (e.g. groups created based on background data) were examined using independent sample t-tests or the non-parametric Mann-Whitney test, in cases of small groups. Differences between rankings in multi-level variables (e.g. place of birth, in a non-dichotomous division) were examined using one-way ANOVA analyses with Tukey posteriori tests or Kruskal Wallis analysis for small groups. Correlations were examined using Pearson correlation analysis. Mediation models using logistics or linear regression were used in attempt to establish possible causality between proved correlations [26, 27]. Finally, comparisons between rankings of different arguments were conducted using MANOVA Repeated Measures analyses, with Bonferroni posteriori tests.

Statistical analysis was done by professional statistics using SPSS Statistics software, Version 22. The study was approved by Bnai-Zion Medical Center Institutional Review Board (Approval Number: 47–15-BNZ).

## Results

Three hundred and twenty-two physicians from specialties that employ endoscopic technics in their practices were surveyed. The mean age was 51.4 (±11) years (range 29–86), 77.3% were men and 68% were born in Israel. Most of the physicians graduated from an Israeli medical faculty and are employed in public hospitals (72.7 and 73%, respectively). Eighty-five specialize in Obstetrics and Gynecology (OBGYN) (26,4%), 65 in Surgery (20.2%), 62 in Gastroenterology (19.3%), 50 in Urology (15.5%), 42 in Ear, Nose and Throat (ENT) and five in Ophthalmology (13%, last two defined as “microsurgery”) and 18 in Orthopedics (5.6%). Full demographic data of study population can be found in the (Additional file 1: Table S1), and specific comparison with the general population of physicians in Israel (based on Ministry of Health data) is shown in Table 1.

**Table 1:** Demographic comparison between study sample and national physicians’ population (based on Health Ministry data)

Specialty	Female Gender (%)		Israeli Medical education (%)		Age over 55 (%)	
	Sample	Population	Sample	Population	Sample	population
Ob-gyn	35.3	39	83.5	65	45.9	59
Surgery	18.8	15	70.3	48	42.2	59
Gastroenterology	29	29	74.2	59	37.1	48
Urology	4	6	44	46	36	61
Ear, nose & throat	17.1	23	85.7	54	34.3	59
Ophthalmology	49	40	57.1	59	14.3	54
Orthopedics	5.6	5	83.3	50	55.6	50
Total	22.7	37 off all specialists	72.7	48 off all specialists	40.30	51.6 off all specialists

**Fig. 1:**
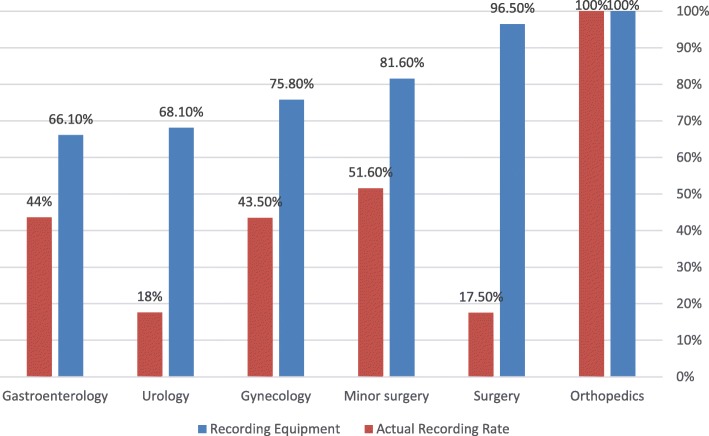
Availability of recording equipment and actual DRD rate by specialties

Eighty-four point 3 % reported performing routine endoscopic procedures. Although 78.2% had the required equipment for digital recording, almost two thirds of them stated that they never or only rarely recorded the procedure (64.2%, vs. 35.8% who always or usually record). As shown in Table 2, the rates of actual routine recording were higher amongst female physicians, physicians who graduated medical faculties in countries other than Israel, and physicians who were involved in a local institute discussion regarding DRD or had clear institutional guidelines on the issue (*p =* 0.043, 0.02, 0.014 and < 0.001, respectively). Statistically significant differences in availability of digital recording equipment and in rates of actual procedure recording were found between specialties (*p* = 0.001 and *p* < 0.001, respectively). The existing gaps between availability of recording equipment and actual DRD rates are demonstrated by Fig. 1.

**Table 2:** Differences in recording habits, by demographic variables and relevant experiences^+^

		Never or Usually not Recording	Alwayes or Usually Recording	P value
Age (years)	(mean ± SD)	51.1 ± 11	49.2 ± 10	0.17
	Age < 55 (N, %)	101 (61.2%)	64 (38.8%)	0.22
	Age > 55 (N, %)	64 (69.5%)	28 (30.5%)	
Gender, N (%)	Male	116 (64.4%)	64 (35.6%)	0.043*
	Female	22 (61.1%)	14 (38.9%)	
Place of birth, N (%)	Israel	95 (65.5%)	50 (34.5%)	0.476
	Other	43 (60.6%)	28 (39.4%)	
Country of Medical Education, N (%)	Israel	107 (68.6%)	49 (31.4%)	0.02*
	Other	31 (51.7%)	29 (48.3%)	
Working Location, N (%)	Tel Aviv	68 (66.0%)	35 (34.0%)	0.534
	Other	70 (61.9%)	43 (38.1%)	
Working Environment, N (%)	Public Hospital	110 (64.3%)	61 (35.7%)	0.76
	Other	28 (62.2%)	17 (37.8%)	
Specialty, N (%)	Gynecology	26 (56.5%)	20 (43.5%)	0.000
	Surgery	47 (82.5%)	10 (17.5%)	
	Gastroenterology	22 (56.4%)	17 (43.6%)	
	Urology	28 (82.4%)	6 (17.6%)	
	Microsurgery	15 (48.4%)	16 (51.6%)	
	Orthopedics	0 (0.0%)	9 (100%)	
Was involved in discussion regarding DD of endoscopy, N (%)	NO	118 (67.8%)	56 (32.2%)	0.014
	YES	20 (47.6%)	22 (52.4%)	
Existing guidelines regarding recording of procedures, N (%)	NO	119 (70.4%)	50 (29.6%)	0.000
	YES	6 (20.7%)	23 (79.3%)	
Was involved** in the “experience” of a lawsuit with a patient in the past, N (%)	NO	87 (64.0%)	49 (36.0%)	0.974
	YES	51 (63.8%)	29 (36.3%)	
TOTAL		(%)	(%)	

Only physicians reported performing routine endoscopies and having the required recording equipment were included in analysis

* Non-significant on multivariate analysis

** Not necessarily personal involvement

**Fig. 2:**
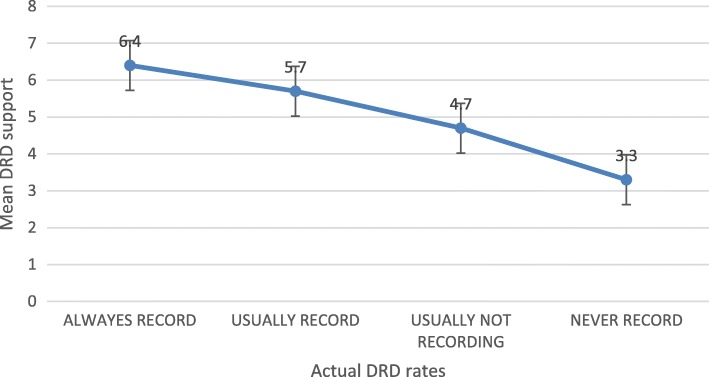
Actual DRD rates and mean DRD support

Average ranking of support of DRD by all participants was 5.07 with a standard deviation of 1.9. No demographic factors were found to correlate with ranking of support (Additional file 1: Table S2). Interestingly, physicians who do ***not*** perform endoscopic procedures in their practices or have no recording equipment, ranked their support of DRD higher than “actual recorders” (5.42 ± 1.7 vs. 4.9 ± 1.9, respectively, *p* = 0.028). Amongst physicians who perform endoscopic procedures and have the required recording equipment, a significant positive correlation exists between actual DRD rates and average support of DRD (*p* < 0.001, Fig. 2).

### Actual DRD rates and support of DRD –mediations models

Following the correlations found between specialty, existence of guidelines, support for recording and actual recording, two mediation models have been observed:

- **Model I** - mediation of the correlation between specialty and actual recording by recording guidelines, and of the correlation between guidelines and actual recording by support for recording. The model was examined for each specialty separately.
- **Model II** - mediation of the correlation between specialty and support for recording by recording guidelines, and of the correlation between guidelines and support for recording by actual recording. The model was examined for each specialty separately.

**Fig. 3:**
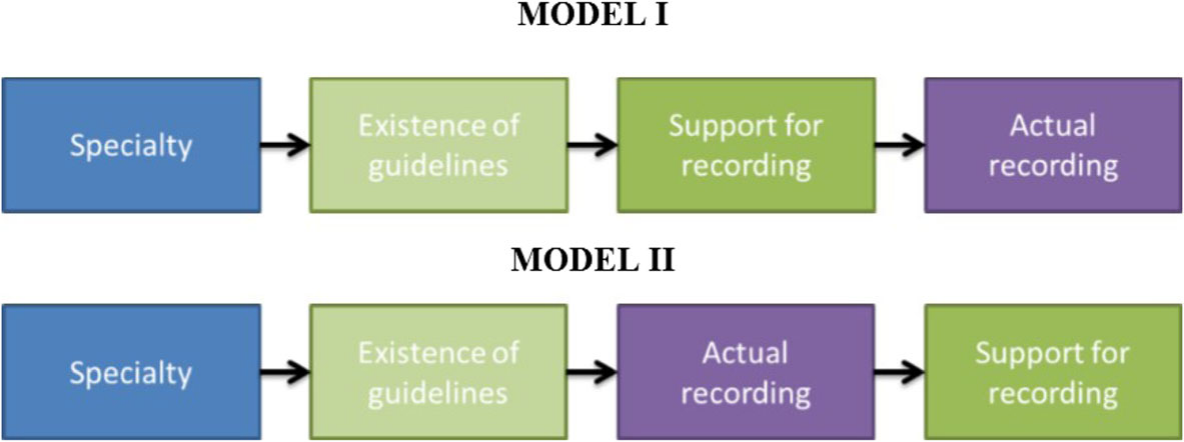
Graphic illustration of the mediation models

The mediation models are graphically illustrated in Fig. 3.

#### Model I results

For the observation of Model I, we employed a stepwise **logistic** regression with an enter method, since the variable “Actual recording” represented a dichotomous response. In the first block, specialty was entered as a dummy variable. In the second block, specialty and guidelines were both entered. In the third block, all three predictors (specialty, guidelines and support for recording) were entered. For each block, the significance of predictors is reported.

For the specialty of surgery, two partial mediations have been found (see Table 3A). Also, the results indicate that being a surgeon decreases the probability for actual recording, while the existence of guidelines and support for recordings increase the probability for actual recording.

**Table 3:** Model I results – Surgery and Urology

A. Surgery specialty					
	B	SE	Wald (df = 1)	Exp(B)	P value
Block 1					
Surgery specialty	−1.30	0.39	11.24	0.27	0.001
Block 2					
Surgery specialty	−1.19	0.41	8.55	0.30	0.003
Recording guidelines	2.12	0.50	18.09	8.34	0.00
Block 3					
Surgery specialty	−1.01	0.43	5.39	0.37	0.02
Recording guidelines	2.24	0.55	16.84	9.40	0.00
Support for recording	0.52	0.11	20.77	1.69	0.00
Block 1 [*χ*^2^(_1)_ = 13.07, *p* < 0.001, Nagelkerke’s *R*^*2*^ = 0.09], block 2 [*χ*^2^_(1)_ = 22.47, *p* < 0.001, Nagelkerke’s *R*^*2*^ = 0.22], and block 3 [*χ*^2^_(1)_ = 28.20, *p* < 0.001, Nagelkerke’s *R*^*2*^ = 0.38] have been found significant.					
B. Urology specialty					
	B	SE	Wald (df = 1)	Exp (B)	P value
Block 1					
Urology specialty	−0.87	0.49	3.19	0.42	0.07
Block 2					
Urology specialty	−0.75	0.51	2.14	0.47	0.14
Recording guidelines	2.17	0.49	19.63	8.79	0.00
Block 3					
Urology specialty	−0.78	0.57	1.87	0.46	0.17
Recording guidelines	2.32	0.54	18.51	10.18	0.00
Support for recording	0.56	0.12	22.80	1.75	0.00
Block 1 [*χ*^2^(_1)_ = 3.58, *p* = 0.06, Nagelkerke’s *R*^*2*^ = 0.03] has been found marginally significant, and block 2 [*χ*^2^_(1)_ = 24.60, *p* < 0.001, Nagelkerke’s *R*^*2*^ = 0.18] and block 3 [*χ*^2^_(1)_ = 31.72, *p* < 0.001, Nagelkerke’s *R*^*2*^ = 0.36] have been found significant.					

For the specialty of urology, a partial mediation was found in the correlation between specialty and actual recording, by the existence of recording guidelines (see Table 3B). It can be observed that being an urologist decreases the probability of actual recording. However, the existence of recording guidelines is a better predictor for actual recording than the urology specialty, and when jointly examined to predict actual recordings, the specialty of urology is not significant (*p* > 0.1).

For the specialties of gastro, gynecology, microsurgery and orthopedics, no mediation has been found. It is important to indicate that guidelines and support for recording have been found as significant predictors of actual recording (Additional file 1: Table S3, panels A-D), so that existence of guidelines and support for recording increase the probability for actual recording.

#### Model II results

For the observation of Model II, we employed a stepwise **linear** regression with an Enter method. All predictors in this model were coded as dummy variables. In the first step, specialty was entered; in the second step, specialty and guidelines were both entered; and in the third step, all three predictors (specialty, guidelines and actual recording) were entered.

For the specialties of surgery and microsurgery, we found a partial mediation in the correlation between specialty and support for recording by actual recording (see Table 4A and B). From the mediation model, it emerges that being a surgeon is negatively correlated with support for recording (*F*_(1,196)_ = 4.24, *p* < 0.05, *R*^2^ = 0.02), while being a micro surgeon is positively correlated with support for recording (*F*_(1,196)_ = 3.04, *p* < 0.1,, *R*^2^ = 0.02). The mediation models also indicate that actual recording is a better predictor of support than the two specialties (surgery or microsurgery) and existence of recording guidelines.

**Table 4:** Model II results – Surgery, Microsurgery, and Orthopedics

A. Surgery specialty					
	B	SE	β	t	P value
Step 1					
Surgery specialty	−0.64	0.31	−0.15	−2.06	0.04
Step 2					
Surgery specialty	−0.59	0.31	−0.13	−1.88	0.06
Recording guidelines	0.47	0.40	0.08	1.18	0.24
Step 3					
Surgery specialty	−0.25	0.30	−0.06	− 0.81	0.42
Recording guidelines	−0.26	0.40	−0.05	−0.66	0.51
Actual recording	1.60	0.30	0.39	5.34	0.00
Step 1 [*F*_(1,196)_ = 4.24, *p* < 0.05, *R*^2^ = 0.02] and step 3 [*F*_(3,194)_ = 11.65, *p* < 0.001, *R*^2^ = 0.14] have been found significant. Step 2 [*F*_(2,195)_ = 2.82, *p* < 0.1, *R*^2^ = 0.02] has been found marginally significant.					
B. Microsurgery specialty					
	B	SE	β	t	P value
Step 1					
Microsurgery specialty	0.69	0.40	0.12	1.74	0.08
Step 2					
Microsurgery specialty	0.72	0.40	0.13	1.83	0.07
Recording guidelines	0.61	0.40	0.11	1.53	0.13
Step 3					
Microsurgery specialty	0.47	0.37	0.08	1.26	0.21
Recording guidelines	−0.20	0.40	−0.04	−0.50	0.62
Actual recording	1.60	0.29	0.39	5.46	0.00
Step 1 [*F*_(1,196)_ = 3.04, *p* < 0.1, *R*^2^ = 0.02] and step 2 [*F*_(2,195)_ = 2.71, *p* < 0.1, *R*^2^ = 0.02] have been found marginally significant. Step 3 [*F*_(3,194)_ = 12.01, *p* < 0.001, *R*^2^ = 0.14] has been found significant.					
C. Orthopedics specialty					
	B	SE	β	t	P value
Step 1					
Orthopedics specialty	1.79	0.67	0.19	2.68	0.01
Step 2					
Orthopedics specialty	1.70	0.67	0.18	2.53	0.01
Recording guidelines	0.46	0.40	0.08	1.15	0.25
Step 3					
Orthopedics specialty	0.81	0.66	0.09	1.24	0.22
Recording guidelines	−0.25	0.40	−0.05	−0.64	0.52
Actual recording	1.55	0.30	0.38	5.14	0.00
Step 1 [*F*_(1,196)_ = 7.16, *p* < 0.01, *R*^2^ = 0.04], step 2 [*F*_(2,195)_ = 4.25, *p* < 0.05, *R*^2^ = 0.03] and step 3 [*F*_(3,194)_ = 12.00, *p* < 0.001, *R*^2^ = 0.14] have been found significant.					

For the specialty of orthopedics, we found a full mediation of the correlation between specialty and support for recording, by actual recording (see Table 4C). The specialty of orthopedics was positively correlated with support.

For the specialties of gastro, gynecology and urology, no mediation has been found. However, actual recording has been found as a significant predictor of support for recording (Additional file 1: Table S4, panels A-C), so that actual recording is positively correlated with support for recording.

### Ranking of specific arguments regarding DRD

**Fig. 4:**
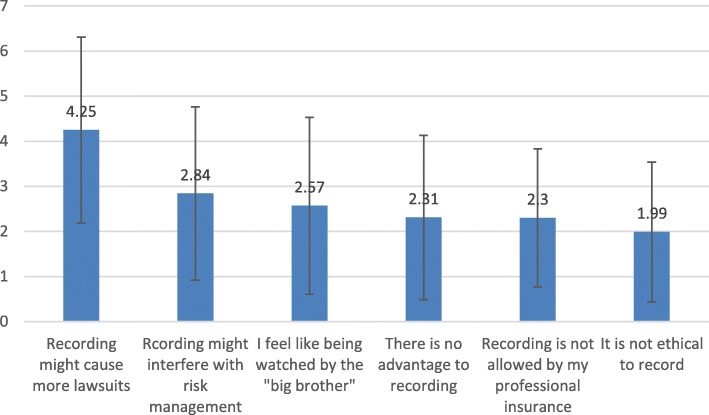
Mean Ratings of Arguments AGAINST Digital Recording (+/−1 S.D.)

**Fig. 5:**
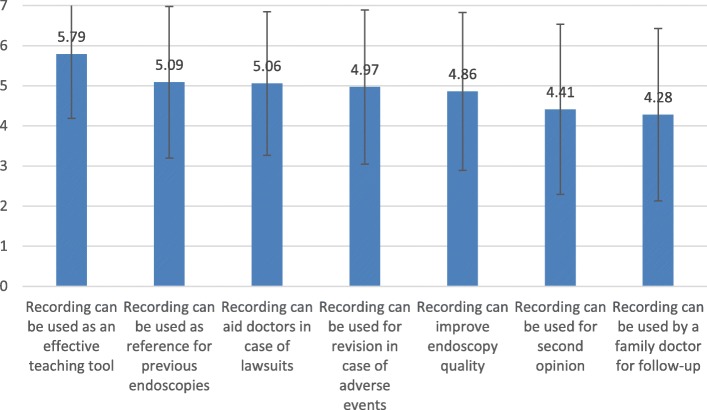
Mean Ratings of Arguments FOR Digital Recording (+/−1 S.D.)

Doctors’ ranking of specific arguments regarding DRD are graphically illustrated in Fig. 4 and Fig. 5. A significant effect of specialty (F _(5,316)_ = 6.03, *p* < 0.001) and a significant effect of argument (F_(5,1580)_ = 85.23, p < 0.001) were found. The argument “Recording might cause more lawsuits” was ranked significantly higher than all other arguments **against** DRD (*p* < 0.001 for all paired comparisons) across specialists. In a Univariate analysis of the specific argument, a significant effect was found for specialty (F _(5,316)_ = 3.19, *p* = 0.008), so that surgeons (M = 4.83 ± 2.19) ranked the argument marginally higher than gynecologists (M = 3.86, SD = 2.22; *p* = 0.058) and microsurgery (M = 3.67 ± 1.82; *p* = 0.060).

The analysis of arguments **for** digital recordings also showed a significant effect of specialty (F _(5,316)_ = 10.07, p < 0.001) and of argument (F_(6,1896)_ = 40.34, p < 0.001). The argument “Recording can be used as an effective teaching tool,” was ranked significantly higher than all other arguments (p < 0.001 for all paired comparisons) across specialists/specialties. In a Univariate analysis of the specific argument, a significant effect was found for specialty (F _(5,316)_ = 8.78, p < 0.001), so that gastro specialists’ rankings of this argument (M = 4.77 ± 0.19) were significantly lower than all other specialists’ rankings.

## Discussion

In this study, we report the results of a large survey aimed to discover “real life” DRD rates amongst physicians in Israel, and their perspectives regarding DRD of endoscopic procedures. We surveyed 322 physicians, from a wide spectrum of specialties that employ endoscopic procedures, including: gastroenterologists, surgeons, gynecologists, microsurgery, urologists and orthopedics.

### General

We found that while the existence of DRD equipment is fairly widespread amongst our sample participants (around 80% for all specialties), actual recording rates vary between specialties. This discrepancy was most prominent for general surgeons (with second highest rate of existence of DRD equipment but lowest rates of actual DRD, compared with of all other specialties), urologists and microsurgery. Univariate analysis showed that being a female physician, being a graduate of a non-Israeli medical faculty, being involved in a discussion regarding DRD or having clear institutional guidelines requiring routine recording, are all associated with higher rates of actual DRD.

### Causality between support and actual recording

The correlation found between DRD rates and physicians’ support of DRD, demonstrated by Fig. 2, seems to be a key finding of this study. Two possible theoretical explanations are suggested for this correlation: the physicians actually highly support DRD and therefore practice it (Model I, the *“logical”* theory), or more interestingly, that routine DRD itself increases support rates (Model II, the *“psychological”* theory). Statistical mediation models were used in order to establish causality (i.e. the “direction” of the correlation) between specialty, guidelines, support and actual recording.

Assuming the “logical” theory (Model I), for all specialties with no exceptions, having institutional guidelines requesting routine recording and a positive support of DRD were found to increase the probability of actual recording. The interesting discovery of this model was that being a surgeon or a urologist decreases actual recording rate despite having guidelines or supporting DRD (but only as a partial mediator). In this model, none of the other specialties mediated the correlation with actual recording.

Assuming the “psychological” theory (Model II), it was found true for all specialties with no exceptions, that actual common practicing of DRD predicted increased support of DRD. As for the different specialties, being a general surgeon correlated with **negative** support of DRD, while being a microsurgeon positively correlated with DRD. Both findings were only partial mediations, which mean that actual recording rates better explain surgeons’ and microsurgeons’ support of DRD. A full mediation was only proved for orthopedics, so it can be concluded that being an orthopedic surgeon is accompanied by higher rates of DRD, followed by increased orthopedics’ support of DRD.

We find Model I to be generally preferred over Model II for two main reasons. First, the R^2^ values for the significant steps in this model were higher (~ 0.38 vs ~ 0.14, see Tables 2 & 3). Secondly, our finding that “non-recorders” (i.e. have no recording equipment or do not practice endoscopic procedures) are more supportive of DRD, contradicts the assumption of Model II that actual recording correlates with positive support of DRD.

### Motivation for recording / refraining

Physicians’ ranking of specific arguments regarding DRD seems to be the second profound finding in our study. All specialties ranked “Recording might cause more lawsuits” significantly higher than all other arguments against DRD, and “Recording could aid teaching of interns” higher than all other arguments in favor of DRD. In other words, physicians were very much concerned about DRD’s potential to enhance lawsuits, and are centrally motivated by litigation concerns. We previously published similar results regarding gastroenterologists’ concerns regarding lawsuits [7] and our current study clearly validates these findings for all other specialties.

As for the second highest ranked argument in favor of DRD, surgeons chose “revision in case of complications.” Interestingly, both gastroenterologists and gynecologists chose the argument “DRD can be helpful in case of lawsuit” as their second highest ranked reason **in favor of** DRD, placing litigatory concerns as central motivations both for and against routinely documenting endoscopic procedures.

litigatory concerns seem to play a stronger deterring role among surgeons, who record significantly less than other specialists, despite extensively possessing DRD equipment.

### Strengths and limitations of research

The strengths of our study include original concept and design, and a relatively large sample size with detailed demographic information, thereby producing a lowered risk of hidden confounders. Additionally, the mediation models we used provide an unusual interpretation of the results.

The limitations of our study include a possible bias as a result of self-reported surveys – especially in terms of the self-selection of those physicians willing to cooperate with such surveys, who might not necessarily represent all endoscopy-performing physicians. Nevertheless, we believe that at least for the major sub specialties we had a large enough sample size to overcome this restriction. Also, the composition of our sample differs from that of the general population in that the sample is more heavily males, Israel educated and mostly younger than 65. Over-representation of males possibly led to minor downward bias in DRD overall rate, while over-representation of Israeli trained physicians probably led to opposite direction bias. In other words, actual DRD use in Israel may be even lower than that in our study. Another possible limitation is the fact that physicians had to choose between specific arguments, which did not necessarily fully reflect their perspectives.

### Policy recommendations

Growing technical capabilities and changes in societal perceptions have (re)ignited the interest of practitioners and health care managers in DRD of medical procedures.

Though the subject has not yet been extensively studied, those studies that have been published have indicated a high potential for benefit from DRD to medical training and practice. Regardless, prevalence of DRD remain comparatively low – both in Israel and worldwide, due to 3 interwoven reasons: a) litigatory concerns – as exemplified in our research; b) lack of pro-DRD policy and c) DRD operational costs.

Our extensive literature review yielded no data regarding specifically drawn governmental directives or regulations nor any relevant professional guidelines, in Israel nor in any other country (save in the Netherlands, as mentioned before [2]). Also, oral Inquiries with heads of two of the above-surveyed Israeli medical associations, revealed that both had no knowledge of such in their corresponding professional associations abroad – which, if proven to be not fully informed, at least comes to show the general low interest in the issue. Similarly, a discussion with representatives from the Department of Risk Management in the Israeli Ministry of Health raised a preference for refraining from issuing instructions on the subject and even a reluctance to endorse DRD systematically.

This policy vacuum is understandable, when viewing the vast writing regarding ethical and legal concerns (as detailed above). It becomes even clearer when considering the still non-negligible costs attributed to medical-grade DRD equipment – due to which the optimal setup for video recording remains uncertain and can vary based on the equipment available at respective institutions. [28]

In light of all the above, as well as the fact that institutional guidelines did show to centrally impact implementation of DRD in our research-sample, we recommend that at this point efforts should be centered around institutional guidelines requiring DRD, where feasible. We also endorse a more thorough, future-anticipating debate, to be conducted within the relevant professional association, as well as the issuing of consequent professional guidelines. We believe that such guidelines should generally encourage DRD, though not presently require it. All this should be followed by extensive educational efforts, aiming to lessen litigatory concerns as well as hands-on exemplifying DRD potential benefits.

## Conclusions

Our current study included almost all the types of physicians in Israel performing any kind of endoscopic procedures (i.e. Orthopedics, Obstetrics and Gynecology, general surgery and more). We found that while DRD facilities and equipment are fairly widespread, actual recording rates are generally low and vary between specialties. Having institutional guidelines requesting routine recording and a positive support of DRD were found to increase the probability of actual recording.

Concerns regarding the effect routine recording might have on litigation outcomes are central to both DRD opposing and DRD supporting positions. Such concerns seem to play a stronger deterring role among surgeons, who record significantly less than other specialists, despite extensively possessing DRD equipment.

These finding should be addressed by educational efforts, centering on professionals from reluctant specialties, as well as by the issuing of professional associations’ and institutional guidelines. Such guidelines should address questions of when, how and under what circumstances DRD should be applied. This should be done in a way that would lessen litigatory concerns while simultaneously increasing realization of the potential benefits of DRD. Further research into the benefits and drawbacks of DRD is also recommended.

## Additional files

Additional file 1: **Table S1.** Study population - demographic data.**Table S2.** Differences in rankings of supporting DRD of endoscopic procedures, based on demographic data.**Table S3.** Model I - Non-significant mediation.**Table S4.** Model II - Non-significant mediation (DOCX 29 kb)

## Data Availability

or analysed /or analysed during the current study are available from the corresponding author on reasonable request. All data generated or analysed during this study are included in this published article [and its supplementary information files].

## Abbreviations

DRD: Digital recording and documentation
