# Supplementary material for: Digital recording and documentation of endoscopic procedures: physicians’ practice and perspectives
Source: Isr J Health Policy Res. 2019 Jul 2;8:57. doi: 10.1186/s13584-019-0332-6 (PMC6607539; doi:10.1186/s13584-019-0332-6)
Supplement: Supplementary file 1 — Table S1. Study population - demographic data.Table S2. Differences in rankings of supporting DRD of endoscopic procedures, based on demographic data.Table S3. Model I - Non-significant mediation.Table S4. Model II - Non-significant mediation (DOCX 29 kb) [file 13584_2019_332_MOESM1_ESM.docx]

**Supplementary Data – DRD of endoscopic procedures**

**TABLE S1: Study population - demographic data**

| Age | mean ± SD, years | 51.4±10.9 |
| --- | --- | --- |
|  | Range, years | 29-86 |
| Gender, N (%) | Male | 249 (77.3%) |
|  | Female | 73 (22.7%) |
| Place of birth, N (%) | Israel | 220 (68.3%) |
|  | Other | 102 (31.7%) |
| Country of Medical Education, N (%) | Israel | 234 (72.7%) |
|  | Other | 88 (27.3%) |
| Work Location, N (%) | Tel Aviv | 158 (49.1%) |
|  | Other | 164 (50.9%) |
| Work Environment, N (%) | Public Hospital | 235 (73%) |
|  | Other | 87 (27%) |
| Specialty, N (%) | Gynecology | 85 (26.4%) |
|  | Surgery | 65 (20.2%) |
|  | Gastroenterology | 62 (19.3%) |
|  | Urology | 50 (15.5%) |
|  | Microsurgery* | 42 (13.0%) |
|  | Orthopedics | 18 (5.6%) |
| **TOTAL** | | 322 (100%) |

*Microsurgery – Ear, Nose and Throat (ENT) and Ophthalmology

| **TABLE S2**: **Differences in rankings of supporting DRD of endoscopic procedures, based on demographic data** | | | | | |
| --- | --- | --- | --- | --- | --- |
|  | | ***Mean*** ± ***SD*** | ***t*** | ***df*** | ***P value*** |
| GENDER | MALE | 5.06±2.01 | 0.08 | 138 | 0.94 |
|  | FEMALE | 5.08±1.68 |  |  |  |
| PLACE OF BIRTH | ISRAEL | 5.04±2.02 | 0.40 | 226 | 0.69 |
|  | OTHER | 5.13±1.74 |  |  |  |
| COUNTRY OF MEDICAL EDUCATION | ISRAEL | 5.04±1.94 | 0.44 | 323 | 0.66 |
|  | OTHER | 5.14±1.94 |  |  |  |
| WORK LOCATION | TEL AVIV | 4.90±2.05 | 1.54 | 323 | 0.13 |
|  | OTHER | 5.23±1.81 |  |  |  |
| WORK ENVIRONMENT | PUBLIC HOSPITAL | 5.09±1.88 | 0.38 | 323 | 0.70 |
|  | OTHER | 5.00±2.09 |  |  |  |

| **TABLE S3**: **Model I - Non-significant mediation**   1. **Gastro specialty** | | | | | | | | | | | | | |
| --- | --- | --- | --- | --- | --- | --- | --- | --- | --- | --- | --- | --- | --- |
|  | | **B** | | | **SE** | | **Wald (df=1)** | | | **Exp(B)** | **P value** | | |
| Block 1 | |  | | |  | |  | |  | |  | | |
| Gastro specialty | | 0.53 | | | 0.37 | | 2.00 | | 1.69 | | 0.16 | | |
| Block 2 | |  | | |  | |  | |  | |  | | |
| Gastro specialty | | 0.16 | | | 0.42 | | 0.14 | | 1.17 | | 0.71 | | |
| Recording guidelines | | 2.18 | | | 0.50 | | 19.27 | | 8.82 | | **0.00** | | |
| Block 3 | |  | | |  | |  | |  | |  | | |
| Gastro specialty | | 0.33 | | | 0.46 | | 0.50 | | 1.39 | | 0.48 | | |
| Recording guidelines | | 2.29 | | | 0.55 | | 17.41 | | 9.86 | | **0.00** | | |
| Support for recording | | 0.56 | | | 0.12 | | 23.26 | | 1.75 | | **0.00** | | |
| Block 2 [*χ^2^*_(1)_=23.99, *p*<0.001, Nagelkerke's *R^2^*=0.17] and block 3 [*χ^2^*_(1)_=32.42, *p*<0.001, Nagelkerke's *R^2^*=0.35] have been found significant. | | | | | | | | | | | | | |
| **B. Gynecology specialty** | | | | | | | | | | | | | |
|  | | **B** | | | **SE** | | **Wald (df=1)** | | | **Exp(B)** | | **P value** | |
| Block 1 | |  | | |  | |  | | |  | |  | |
| Gynecology specialty | | 0.30 | | | 0.36 | | 0.68 | | | 1.35 | | 0.41 | |
| Block 2 | |  | | |  | |  | | |  | |  | |
| Gynecology specialty | | 0.33 | | | 0.39 | | 0.74 | | | 1.39 | | 0.39 | |
| Recording guidelines | | 2.22 | | | 0.49 | | 20.53 | | | 9.19 | | **0.00** | |
| Block 3 | |  | | |  | |  | | |  | |  | |
| Gynecology specialty | | 0.23 | | | 0.410 | | 0.31 | | | 1.26 | | 0.58 | |
| Recording guidelines | | 2.36 | | | 0.54 | | 19.09 | | | 10.57 | | **0.00** | |
| Support for recording | | 0.55 | | | 0.12 | | 22.87 | | | 1.74 | | **0.00** | |
| Block 2 [*χ^2^*_(1)_=25.88, *p*<0.001, Nagelkerke's *R^2^*=0.17] and block 3 [*χ^2^*_(1)_=31.64, *p*<0.001, Nagelkerke's *R^2^*=0.35] have been found significant. | | | | | | | | | | | | | |
| **C. Microsurgery specialty** | | | | | | | | | | | | | |
|  | | | **B** | | **SE** | | **Wald (df=1)** | | | **Exp(B)** | | **P value** | |
| Block 1 | | |  | |  | |  | | |  | |  | |
| Microsurgery specialty | | | 0.55 | | 0.41 | | 1.87 | | | 1.74 | | 0.17 | |
| Block 2 | | |  | |  | |  | | |  | |  | |
| Microsurgery specialty | | | 0.74 | | 0.43 | | 3.00 | | | 2.09 | | **0.08** | |
| Recording guidelines | | | 2.27 | | 0.49 | | 21.31 | | | 9.73 | | **0.00** | |
| Block 3 | | |  | |  | |  | | |  | |  | |
| Microsurgery specialty | | | 0.48 | | 0.46 | | 1.12 | | | 1.62 | | 0.29 | |
| Recording guidelines | | | 2.39 | | 0.54 | | 19.51 | | | 10.91 | | **0.00** | |
| Support for recording | | | 0.54 | | 0.12 | | 22.13 | | | 1.72 | | **0.00** | |
| Block 2 [*χ^2^*_(1)_=26.92, *p*<0.001, Nagelkerke's *R^2^*=0.19] and block 3 [*χ^2^*_(1)_=30.24, *p*<0.001, Nagelkerke's *R^2^*=0.35] have been found significant. | | | | | | | | | | | | | |
| **D. Orthopedics specialty** | | | | | | | | | | | | | |
|  | **B** | | | **SE** | | **Wald (df=1)** | | **Exp (B)** | | | | | **P value** |
| Block 1 |  | | |  | |  | |  | | | | |  |
| Orthopedics specialty | 21.87 | | | 13397.66 | | 0.00 | | 3155224345.56 | | | | | 1.00 |
| Block 2 |  | | |  | |  | |  | | | | |  |
| Orthopedics specialty | 21.69 | | | 12360.21 | | 0.00 | | 2616615282.94 | | | | | 1.00 |
| Recording guidelines | 2.20 | | | 0.50 | | 19.51 | | 9.02 | | | | | **0.00** |
| Block 3 |  | | |  | |  | |  | | | | |  |
| Orthopedics specialty | 21.01 | | | 12385.67 | | 0.00 | | 1329631933.78 | | | | | 1.00 |
| Recording guidelines | 2.35 | | | 0.54 | | 18.62 | | 10.48 | | | | | **0.00** |
| Support for recording | 0.51 | | | 0.12 | | 19.32 | | 1.66 | | | | | **0.00** |
| Block 1 [*χ^2^*_(1)_=18.70, *p*<0.001, Nagelkerke's *R^2^*=0.12], block 2 [*χ^2^*_(1)_=23.75, *p*<0.001, Nagelkerke's *R^2^*=0.26], and block 3 [*χ^2^*_(1)_=25.86, *p*<0.001, Nagelkerke's *R^2^*=0.40] have been found significant. | | | | | | | | | | | | | |

**TABLE S4**: **Model II - Non-significant mediation**

| **A. Gastro specialty** | | | | | |
| --- | --- | --- | --- | --- | --- |
|  | **B** | **SE** | **β** | **t** | **P value** |
| Step 1 |  |  |  |  |  |
| Gastro specialty | -0.15 | 0.37 | -0.03 | -0.42 | 0.67 |
| Step 2 |  |  |  |  |  |
| Gastro specialty | -0.28 | 0.37 | -0.05 | -0.74 | 0.46 |
| Recording guidelines | 0.64 | 0.41 | 0.11 | 1.56 | 0.12 |
| Step 3 |  |  |  |  |  |
| Gastro specialty | -0.33 | 0.35 | -0.06 | -0.95 | 0.34 |
| Recording guidelines | -0.17 | 0.41 | -0.03 | -0.43 | 0.67 |
| Actual recording | 1.66 | 0.29 | 0.40 | 5.68 | **0.00** |
| Step 3 [*F*_(3,194)_=11.75, *p*<0.001, *R*^2^=0.14] has been found significant. | | | | | |
| **B. Gynecology specialty** | | | | | |
|  | **B** | **SE** | **β** | **t** | **P value** |
| Step 1 |  |  |  |  |  |
| Gynecology specialty | 0.30 | 0.35 | 0.06 | 0.85 | 0.39 |
| Step 2 |  |  |  |  |  |
| Gynecology specialty | 0.30 | 0.35 | 0.06 | 0.85 | 0.40 |
| Recording guidelines | 0.57 | 0.40 | 0.10 | 1.43 | 0.15 |
| Step 3 |  |  |  |  |  |
| Gynecology specialty | 0.19 | 0.33 | 0.04 | 0.57 | 0.57 |
| Recording guidelines | -0.24 | 0.40 | -0.04 | -0.61 | 0.54 |
| Actual recording | 1.64 | 0.29 | 0.40 | 5.60 | **0.00** |
| Step 3 [*F*_(3,194)_=11.52, *p*<0.001, *R*^2^=0.14] has been found significant. | | | | | |
| **C. Urology specialty** | | | | | |
|  | **B** | **SE** | **β** | **t** | **P value** |
| Step 1 |  |  |  |  |  |
| Urology specialty | -0.49 | 0.41 | -0.09 | -1.22 | 0.22 |
| Step 2 |  |  |  |  |  |
| Urology specialty | -0.45 | 0.41 | -0.08 | -1.10 | 0.27 |
| Recording guidelines | 0.53 | 0.40 | 0.10 | 1.34 | 0.18 |
| Step 3 |  |  |  |  |  |
| Urology specialty | -0.22 | 0.38 | -0.04 | -0.59 | 0.56 |
| Recording guidelines | -0.26 | 0.40 | -0.05 | -0.64 | 0.52 |
| Actual recording | 1.63 | 0.29 | 0.40 | 5.55 | **0.00** |
| Step 3 [*F*_(3,194)_=11.53, *p*<0.001, *R*^2^=0.14] has been found significant. | | | | | |
